# Acupuncture for Anxiety Disorders: A Systematic Review

**DOI:** 10.1192/j.eurpsy.2026.11221

**Published:** 2026-07-31

**Authors:** M. F. Zeilstra, M. Arts, K. M. Zeilstra-Kalt, L. de Jonge

**Affiliations:** 1 Integrative Medicine, Center de Wetering, Leeuwarden; 2 Department of Geriatric Psychiatry and Neuropsychiatry, GGZ-WNB, Halsteren; 3 Integrative Medicine, Leonardo Scientific Research Institute, Groningen, Netherlands

## Abstract

**Introduction:**

Anxiety disorders are among the most prevalent mental health conditions worldwide and are associated with significant personal and societal burden. Conventional treatments, including pharmacotherapy and cognitive-behavioral therapy, are effective but not always accessible, well-tolerated, or sufficient. Acupuncture, a core component of traditional Chinese medicine, has been investigated as a complementary treatment for anxiety. This systematic review evaluates the evidence on the effectiveness and mechanisms of acupuncture in alleviating anxiety symptoms.

**Objectives:**

The aims of this review are to: (a) synthesize available clinical evidence on acupuncture for anxiety disorders, (b) evaluate its efficacy compared with standard treatments and sham procedures, and (c) discuss potential biological and psychological mechanisms underlying observed effects.

**Methods:**

A systematic search was conducted in PubMed, EMBASE, PsycINFO, and Cochrane Library for randomized controlled trials (RCTs). Studies were included if they assessed the impact of acupuncture on clinically diagnosed anxiety disorders or validated anxiety scales.

**Results:**

Acupuncture demonstrated a moderate reduction in anxiety symptoms compared with baseline and sham interventions. Some studies reported comparable effectiveness to pharmacological treatments, with fewer adverse effects. Evidence suggests enhanced efficacy when acupuncture was used as an adjunct to conventional therapy. Proposed mechanisms include modulation of the hypothalamic–pituitary–adrenal (HPA) axis, regulation of autonomic nervous system balance, increased release of endogenous opioids and serotonin, and reduction of inflammatory markers. However, heterogeneity in study design, sample size, and acupuncture protocols limits the strength of conclusions.

**Conclusions:**

Current evidence indicates that acupuncture may provide clinically meaningful benefits for individuals with anxiety disorders, particularly as an adjunctive treatment. While findings are promising, larger, rigorously designed RCTs with standardized protocols are needed to confirm efficacy, establish optimal treatment parameters, and clarify underlying mechanisms.

**Disclosure of Interest:**

None Declared

